# Cross-cultural differences in resolving sacrificial dilemmas: choices made and how they relate to judgments of their social acceptability

**DOI:** 10.3389/fpsyg.2025.1448153

**Published:** 2025-04-15

**Authors:** Xinyu Jiang, Nigel Harvey

**Affiliations:** University College London, London, United Kingdom

**Keywords:** sacrificial dilemma, ethical choice, ethical acceptability, cultural differences, moral judgment

## Abstract

Samples of English and Chinese people judged the likelihood that they would sacrifice the life (or health) of one person to save the life (or health) of five people by performing an impersonal action (flipping a switch) or a personal one (pushing someone over a bridge). They also judged how many people out of 100 would consider their choice to be morally acceptable. Judgments by people in the two cultures were similar in two ways. First and consistently with previous work, people in both groups were more likely to sacrifice one life to save five when the action was impersonal; however, they were no more likely to make that sacrifice to save the health of five people than to save the lives of those people. Second, the likelihood of people in both cultures deciding on a sacrificial action was less than their assessments of the likelihood that such an action was morally acceptable, a result that is the opposite of what has been previously found. This contrast can be explained by recognizing the difference between asking people to assess how acceptable moral choices are to participants themselves (previous reports) and asking them to judge how acceptable those choices are to other people (this report). The two cultures also differed in two ways. Chinese participants (a) showed a larger difference between the likelihood of people acting and their assessments of the likelihood that acting would be acceptable to others, and (b) were less likely to act in impersonal dilemmas. These cross-cultural differences imply that Chinese participants were more influenced by their judgments of what other people would think about sacrificial action.

## Introduction

Sacrificial dilemmas are used to explore how people make ethical judgments. One example is the trolley dilemma, inspired by Foot (1967): a trolley is heading toward five individuals working on the main track who will be killed if nothing is done; an agent must decide whether to flip a switch to redirect the trolley to a side-track where it would kill one person who is working there.^1^ Choosing to sacrifice one person for the greater good reflects consequentialist (e.g., utilitarian) thinking (Mill, 1998/1868). Refraining from such action aligns with deontological thinking because it indicates use of the moral principle of not harming others (Kant, 1998/1785). Researchers use this dilemma to identify factors that influence ethical decision-making.^2^ In this study of cross-cultural differences in ethical judgments, we focus on three of these factors.

Thomson (1985) introduced the footbridge variant of the dilemma: here the agent is standing on a footbridge over the main track and must decide whether to push an overweight person over the bridge into the path of the trolley, an action that would save the five workers on the track but kill the overweight person. People are less likely to act in this version of the dilemma than in the original side-track version (Greene et al., 2009; Hauser et al., 2007). Why is that? Whereas the original side-track version of the dilemma is impersonal because the agent indirectly harms the individual as a side-effect of their action, this footbridge version is a personal one: the agent directly and intentionally harms the overweight person by pushing them over the footbridge.

According to dual-system theories (e.g., Kahneman, 2011), information processing is carried out either by a fast, intuitive, emotional system (System 1) or by a slower deliberative, cognitive system (System 2). Greene et al. (2001) argued that System 1 plays a more significant role in personal dilemmas but System 2 becomes more prominent in impersonal dilemmas. The resulting greater emotional arousal in the processing of personal dilemmas deters people from making decisions to actively sacrifice the single individual in those dilemmas. This produces the observed difference in the way the two versions of the trolley dilemma are resolved.^3^

### Cross-cultural comparisons

Recently, there has been increased interest in whether people from different cultures respond to sacrificial dilemmas in similar ways. Gold (2023) reviewed 14 studies of this issue and concluded: “There is not a great deal of evidence on cross-cultural variation in response to trolley problems and it is rather mixed, but a preliminary inspection suggests that it is plausible that cross-cultural differences in judgments exist” (p 210). Awad et al. (2020) supported this claim. They compared responses to three different types of dilemma, comprising the side-track and footbridge dilemmas that we outlined above and a third type of trolley problem, the loop dilemma. They found that responses to these dilemmas were ordered in the same way in each of 42 different countries: the ordering of dilemmas was universal. However, the acceptability of sacrifice showed considerable variation across countries: specifically, it was significantly lower in Eastern countries than in Western ones. Bago et al. (2023) report a similar study on participants drawn from 45 different countries and obtained results broadly consistent with those of Awad et al. (2020).

Thus, up to now, findings indicate that *ordering* of acceptability of sacrifice across different dilemma types (e.g., personal, impersonal) is universal; Awad et al. (2020) suggest (without supporting evidence) that this implies that it arises from basic cognitive processes rather than from use of cultural norms. However, the *level* of acceptability of sacrifice in all those dilemmas varies between Eastern and Western participants; this implies it is determined by cultural norms. What other factors have universal effects on responses to sacrificial dilemmas and what other factors have culture-specific effects?

In what follows, we examine effects of three factors. The first of these is *dilemma type* (personal versus impersonal); here we expect to replicate the effects reported by Awad et al. (2020) and Bago et al. (2023). The ordering of the acceptability of sacrifice over different dilemmas should be the same in different cultures.

The second factor that we examine is the *severity of consequences* associated with different outcomes. Although most studies have focused on how willing people are to sacrifice one life to save a number of other lives, the conclusions derived from these studies are often generalized to those that do not involve lives. For example, researchers have utilized research involving trolley dilemmas to predict vaccination decisions (Oftedal et al., 2020), even though vaccinations do not typically cause deaths (Miller et al., 2015).

Greene et al. (2004) showed that increased emotional arousal leads to ethical judgments that are more likely to be deontological. If dilemmas associated with more severe consequences evoke stronger emotions, people’s ethical judgments when faced with such dilemmas will be more likely to rely on deontological reasoning than dilemmas associated with less severe consequences (e.g., injury). We know that East Asian participants tend to make more use of deontological reasoning when solving sacrificial dilemmas (e.g., Ahlenius and Tännsjö, 2012). As a result, we may find that the magnitude of any effects of this factor are culture-specific.

The third factor that we investigate is *measurement type*. Two types of questions are commonly used to assess moral decisions. The first focuses on moral judgment by asking people to evaluate the ethical acceptability of an action. The second deals with moral behavior by asking people about what their choice of action would be. Studies have revealed that individuals tend to make utilitarian choices of actions in trolley dilemmas while holding less utilitarian assessments of the acceptability of such choices (Gold et al., 2015; Kurzban et al., 2012; Tassy et al., 2013). Such findings indicate a disparity between ethical judgment and ethical behavior, indicating that individuals do not always align their behavior with their judgments. Given that Chinese people respond more deontologically when making ethical decisions (Ahlenius and Tännsjö, 2012), they may show a reduced disparity between ethical judgment and ethical behavior.

### Ethical acceptability of sacrificial actions

The third factor specified above is concerned with an observed difference between the likelihood that people will sacrifice one individual to save a number of others and judgments of the ethical acceptability of such sacrificial actions. Studies have shown that people tend to make utilitarian choices while holding less utilitarian assessments of such choices (Gold et al., 2015; Kurzban et al., 2012; Tassy et al., 2013). In all these cases, researchers have measured how ethically acceptable the participants themselves judge the sacrificial action to be. People are asked to assess how ethically acceptable the sacrificial action is to them personally.

However, we know that what people decide to do when faced with sacrificial dilemmas is influenced by other people’s opinions. For example, Kundu and Morris elicited conformity effects in the ethical domain by using objective consensus information within a paradigm akin to that used by Asch (1956). Furthermore, in a series of dilemmas, Bostyn and Roets (2017) found that participants strongly conformed to deontological majorities but exhibited less conformity to consequentialist majorities. They hypothesized that this asymmetry in conformism is attributable to people’s general perceptions of consequentialist individuals as untrustworthy (Everett et al., 2016) and lacking empathy and moral character (Uhlmann et al., 2013). Hence, it makes sense to measure people’s judgments of the social acceptability of their ethical choices. This is because those judgments may influence the expression of their own ethical opinions.

In our experiments, we asked participants to judge how many people out of a group of 100 would judge the action described in the dilemma as morally acceptable. If people fully conform to what they judge other people’s opinions to be, we would expect their assessments of the social acceptability of a sacrificial action to be broadly similar to their own personal judgments of the acceptability of that action. In other words, we expect broadly similar results to those obtained with personal assessments of ethical acceptability by Gold et al. (2015), Kurzban et al. (2012), and Tassy et al. (2013). That is to say that people will make utilitarian choices while judging that other people would resolve sacrificial dilemmas in a less utilitarian manner.

## Rationale for studies

Research on effects of one of these factors (dilemma type) is well-established. However, work on the other two factors (severity of consequences, type of measurement) is sparse. Furthermore, there appears to be no research on how effects of the different factors interact. Thus, before examining whether their effects differ across cultures, we decided that it would be wise to establish whether the expected effects described above exist within a single culture.

Based on the work reviewed above, we expected that ethical judgments would be more utilitarian for impersonal than for personal dilemmas (H_1_), that those judgments would be more deontological for dilemmas involving deaths than for those involving no-life-changing harm (H_2_), and that judgments of the acceptability of ethical choices would be less utilitarian than the choices themselves (H_3_). We also explored whether effects of these three factors interact but we had no specific hypotheses about the nature of any such interactions.

Our first study was carried out with English participants because previous work on which the above hypotheses are based used Western participants. The results from this study were not all as expected. Despite this, we carried out a second study on Chinese participants using exactly the same design. Our hypothesis (H_4_) was that the findings from our first study would be replicated but with all means shifted away from utilitarianism (Ahlenius and Tännsjö, 2012). The same pattern of results obtained with English participants in Study 1 was indeed found again with Chinese participants in Study 2. However, a direct statistical comparison of the results of the two studies to determine whether the effects of the three factors were of the same magnitude in the two cultures revealed additional unexpected findings.

## Experiment **1**

This experiment tested the above three hypotheses on a sample of English participants.

### Method

This was an online study. It used the trolley dilemma scenarios developed by Gold et al. (2013) but modified so that the agent in each scenario was changed from a man named ‘Peter’ to ‘You’. Ethical approval (EP/2016/003) was provided by the Department of Experimental Psychology of University College London.

#### Participants

One hundred and seven participants were recruited via Prolific^4^ and were paid £1.00 for their participation. Of these, seven had passed through the Prolific filters (i.e., their native language was English and they lived in the UK) but were excluded because their cultural background was not English. A further six participants were excluded because they failed the attention check (described below). As a result, the final sample comprised 94 participants, 46 of whom were male and 48 of whom were female. Their mean age was 40 years, with a standard deviation of 15 years. The majority of them (83%) were educated to the level of a Bachelor degree or above.

To be considered to be culturally English, participants had to have English as their first language, had to be born in England, and had to have spent most of their lives in England. These criteria excluded participants from other parts of the British Isles where other languages are spoken (e.g., Welsh, Scots Gaelic, Irish Gaelic, Manx Gaelg).

#### Stimulus materials

All materials were presented to participants in English. Four trolley dilemmas of different types (impersonal, personal) and different degrees of consequence severity (death, injury) were presented to each participant. Scenarios were as follows for impersonal dilemmas with variations used to manipulate severity of consequences shown in bold.

*You are standing by the railroad tracks when you notice a train rolling out of control. It is moving so fast that anyone it hits will **die**/ **be severely injured but with no life-changing consequences**. Ahead on the main track are five people. There is one person standing on a side track that does not re-join the main track. If you do nothing, the train will hit the five people on the main track, but not the one person on the side track. If you flip a switch next to you, it will divert the train to the side track where it will hit the one person, and not hit the five people on the main track.*

For personal dilemmas, the text was as follows.

*You are standing on a footbridge over the railroad tracks when you notice a train rolling out of control. It is moving so fast that anyone it hits will **die**/ **be severely injured but with no life-changing consequences**. Ahead on the track are five people. There is a large person standing near you on the footbridge, and this large person weighs enough that the train would slow down if it hit him (you do not weigh enough to slow down the train). If you do nothing, the train will hit the five people on the track. If you push the one person, that one person will fall onto the track, where the train will hit the one person, slow down because of the one person, and not hit the five people farther down the track.*

For each dilemma, participants gave two responses on a 0–100 scale. First, they indicated their perception of the ethical acceptability of action by estimating the number of people out of 100 who would judge the action described in the dilemma as morally acceptable. Second, they gave the likelihood (0–100%) that they would act themselves.

To check that participants were attending, an attention check was run after they had responded to the four scenarios. This were required them to answer a counter-intuitive question (“What is the common color of the sky?”) with the response “Green” rather than with “Blue,” “Yellow,” or “Red.” Failure to answer this question correctly resulted in exclusion of the participant from the data analysis.

#### Design

The experiment used a within-participant three-way factorial design. The factors were action type (impersonal/personal), severity of consequences (death/injury), and measurement type (judged moral acceptability versus judged likelihood of acting). Order of presentation of the four scenarios was randomized separately for each participant. For each scenario, participants first judged the moral acceptability of acting and then assessed their own likelihood of acting.

#### Procedure

The experiment was run online using Gorilla (Anwyl-Irvine et al., 2020). People were first informed about the experiment, told that it had been given the ethical permission, and asked for their consent to participate. After giving their consent, they received their instructions and then responded to the four scenarios. They then completed the attention check and filled in the demographic questionnaire. Finally, they were debriefed and paid.

### Results

**Figure 1:**
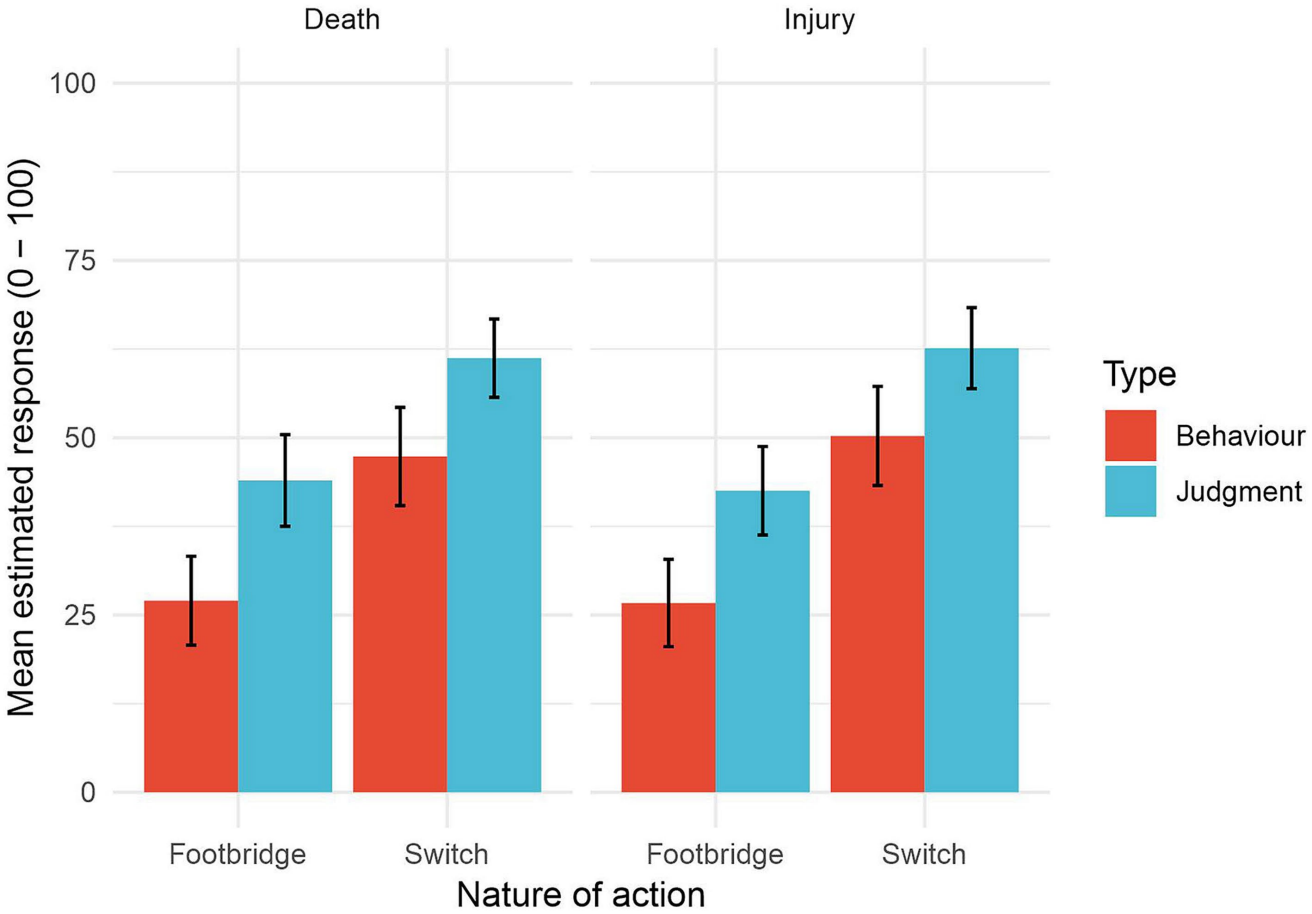
Experiment 1: Mean judgments of moral acceptability (labeled Judgment) and likelihood of acting (labeled Behavior) for each of the four dilemma types. (Error bars show 95% confidence intervals.)

A three-way within-participant ANOVA^5^ showed only main effects of action type (*F*(1, 93) = 71.54, *p* < 0.001, *η*^2^ = 0.097) and measurement type (*F*(1, 93) = 72.49, *p* < 0.001, *η*^2^ = 0.054). Participants’ responses were more utilitarian for side-track dilemmas than footbridge dilemmas and their judgments of the moral acceptability of acting in scenarios were more utilitarian than their judgments that they themselves would act in those scenarios. Thus data (Figure 1) are consistent with one of our hypotheses (H_1_), are in the opposite direction to that expected for another (H_3_), and show no difference where one was expected for the remaining one (H_2_).

### Discussion

One of the effects that we obtained is consistent with what we expected from previous reports. The greater tendency toward action in side-track dilemmas than in footbridge dilemmas is well-documented (e.g., Greene et al., 2009; Hauser et al., 2007). We expected to find it here (H_1_) and we did. Greene et al. (2004) argued that this effect arises because personal dilemmas, such as the footbridge dilemma, lead to emotional arousal and that this, in turn, causes people to rely more on deontological reasoning.

As it is reasonable to expect that dilemmas with more severe consequences should lead to greater emotional arousal, we expected that such dilemmas would produce a greater tendency toward deontological judgments (H_2_). However, contrary to this expectation, our data show no evidence that consequence severity affects ethical decision-making. There are two precedents for this unexpected finding.

First, Waldmann and Dieterich (2007) examined ethical judgments using dilemmas that involved consequences of different levels of severity (e.g., death versus paraplegia). Their results indicated that individuals were less likely to act in personal than in impersonal dilemmas irrespective of the severity of the outcomes. In other words, increased outcome severity that was likely to elicit stronger emotions was not found to increase deontological responding to personal dilemmas. Second, Gold et al. (2013) studied trolley dilemmas involving consequences of different levels of severity, including death, limb loss, broken bones, and bruising. Regardless of the severity of the consequences, participants consistently rated the action as more morally correct in the side-track version than in the footbridge version of the dilemma. These studies suggest that ethical decision-making in sacrificial dilemmas may indeed be independent of the severity of the consequences in those dilemmas.

Why should this be so? In explaining their own failure to obtain an effect of outcome severity, Gold et al. (2013) point out that Greene et al.’s (2004) dual process theory makes no explicit prediction about whether severity of outcome should affect ethical judgment. (However, if an effect had been found, their theory could be extended to account for it.) In contrast, Gold et al. (2013) point out that Mikhail’s (2011) theory that we possess a Universal Moral Grammar, which takes input from moral dilemmas and uses a set of deontic rules to classify actions into either ethically permissible or impermissible, does make an explicit prediction. Mikhail (2011, p133) argues that both battery and homicide are interpreted by the grammar as impermissible: “Any normative system seeking to achieve descriptive adequacy … must include or otherwise account for a small number of absolute or near-absolute prohibitions against various forms of trespass, such as battery, assault, rape, murder, fraud, deceit, and so on.” For Gold et al. (2013, p230), this means that “according to the Universal Moral Grammar theory, it should not make a difference to people’s moral intuitions if death is replaced with bodily injury.”

We can also ask whether manipulating outcome severity within participants rather than between participants could have contributed to our failure to observe an effect of this variable. Within-participant designs make the variables that are being examined in an experiment more salient to participants than between-participant designs do. As a result, the difference between the outcome severity of death and severe injury should be easier rather than harder to evaluate in a within-participant design (Hsee, 1996). Hence it is *less* likely that an effect of outcome severity would have be obtained if this variable had been manipulated between participants.

With respect to H_3_, there was an effect of measurement type but its direction was the opposite of what we had expected: judgments of acceptability of ethical choices were *more* utilitarian than the choices themselves. On average, people acted within the range of what they judged to be acceptable to other people. In contrast, previous studies have found that people were willing to act even in situations in which they personally considered acting to be unethical. For example, Tassy et al. (2013) found that the probability of people choosing to act was 51% when the probability that they judged acting to be acceptable was just 43%. Similarly, Gold et al. (2015) found that 85% of people (averaged across side-track and footbridge conditions) said that they would act whereas only 65.1% of them considered it permissible to act. Finally, Kurzban et al. (2012) found that 52.5% of people (averaged across side-track and footbridge conditions) said that they would act even though 65.7% of them considered that it would be wrong to do so. Before discussing the reasons for these unexpected results, we will examine whether they are replicated in a sample of participants drawn from a different culture.

## Experiment 2

Like most studies of ethical judgment, our first experiment used participants drawn from a Western culture. This second experiment was designed to determine whether our findings would generalize across cultures. In particular, we tested Chinese participants because there is evidence indicating that such people make ethical judgments that are more deontological than Western participants (Ahlenius and Tännsjö, 2012; Awad et al., 2018; Gold et al., 2014). Replication of the effects reported in Experiment 1 could still be observed in cultures that generally respond in a more deontological manner than Westerners. For this to happen, those effects would be preserved but judgments would be made lower on the response scales than in Experiment 1.

### Method

Experiment 2 repeated the first experiment but with Chinese participants and with stimulus materials translated into Mandarin Chinese. The experiment was again performed online and it was analyzed in the same way as before to test the same three hypotheses as the first experiment. To replicate the earlier results, we expected data to be consistent with H_1_ but not with H_2_ or H_3_. After presenting the results of the experiment, we directly compare the results in the two experiments using a four-way mixed ANOVA using culture as a between-participants factor. In addition to replicating the main effects of Action Type (personal, impersonal) and Measurement Type (Perception of Acceptability of Acting, Likelihood of Acting), we expected this to produce a main effect of culture showing that Chinese participants respond in a less utilitarian manner than English participants (H_4_).

Design and procedure were identical to those reported for Experiment 1.

#### Participants

Fifty-nine participants were recruited via Prolific and 61 were recruited via snowball sampling. Of these, 15 were excluded because they failed the attention check and 11 were excluded because they were not culturally Chinese. This left a sample of 94 participants (30 men, 62 women, two non-binary gender) who were entered into the analysis. Their mean age was 27 years (SD: 9 years) and 95% of them had a Bachelor degree or above.

To be considered to be culturally Chinese, participants had to have a Chinese language as their first language, had to be born in mainland China, Hong Kong or Taiwan, and had to have spent most of their lives in one of those places. These criteria did not exclude people who may have spent some time living temporarily outside China (e.g., as holiday makers or as students).

#### Stimulus materials

People adopt a more utilitarian approach when responding to trolley dilemma scenarios in a second language (Costa et al., 2014). To make valid cross-cultural comparisons, materials should be presented in participants’ first language. Hence the materials used in Experiment 1 were translated into Mandarin by a competent bilingual speaker. After initial translation, they were back-translated into English by another competent bilingual speaker. The results were compared with the original, adjustments made where necessary, and the process repeated until the original and back-translated version were indistinguishable.

### Results

**Figure 2:**
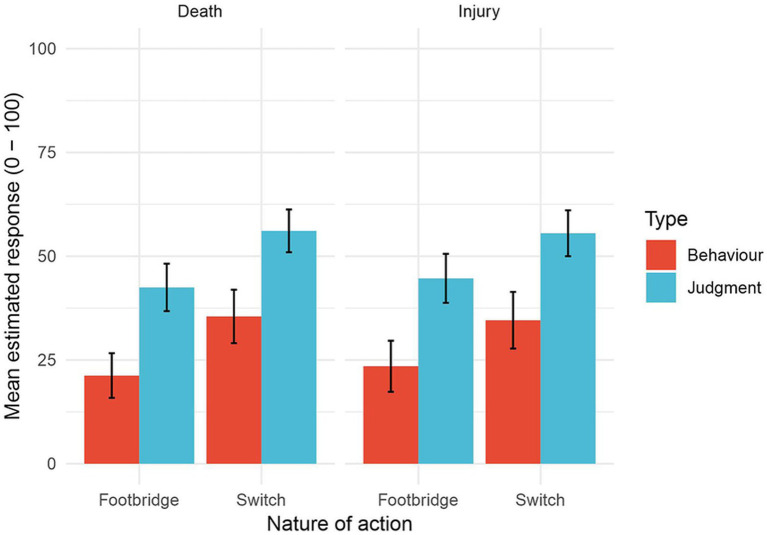
Experiment 2: Mean judgments of moral acceptability (labeled ‘Judgment’) and likelihood of acting (labeled ‘Behavior’) for each of the four dilemma types. (Error bars show 95% confidence intervals.)

Data for each of the eight conditions in the experiment are shown in Figure 2. As in Experiment 1, they show that action type and measurement type but not consequence severity influence people’s judgments. Again, except for acceptability judgments in impersonal dilemmas, judgments were not utilitarian (i.e., above 50) overall.

A three-way within-participant ANOVA^6^ showed significant main effects only for action type (*F* (1, 93) = 30.70, *p* < 0.001, *η*^2^ = 0.044) and measurement type (*F* (1, 93) = 69.77, *p* < 0.001, *η*^2^ = 0.115). Participants’ responses were more utilitarian for side-track dilemmas than footbridge dilemmas and their judgments of the moral acceptability of acting in scenarios were more utilitarian than their judgments that they themselves would act in those scenarios. Thus data (Figure 2) are again consistent with H_1_ but not with H_2_ or H_3_.

### Discussion

Results of this second experiment replicated those of the first. We again obtained results consistent with H_1_: responses were more utilitarian in side-track than in footbridge dilemmas. As before, there was no evidence of an effect of severity of consequences (H_2_). Finally, the effect of measurement type was again exactly the opposite of what we expected on the basis of previous work (H_3_).

We now consider possible reasons for the reversal in our experiments of previously reported effects of measurement type. We asked for judgments of ethical acceptability of acting in each dilemma before we asked participants whether they themselves would act in that dilemma. It is possible that ordering responses in this way prompted participants to ensure that their judgments of their likelihood of acting were no greater than their judgments of the probability that action would be considered acceptable. However, this explanation for our findings is not adequate. In Tassy et al.’s (2013) experiment, responses were ordered in the same way as they were ordered in our experiments yet their results were the opposite of ours.^7^

A better explanation for the reversal in our experiments of the previously reported effects of measurement type is based on a difference in the sort of ethical acceptability that participants judged. In previous studies, people assessed how ethically acceptable they personally considered acting to be. For example, Gold et al. (2015) asked participants to assess the rightness of acting on a nine-point scale; Kurzban et al. (2012) asked them to judge whether acting was morally wrong; Tassy et al. (2013) asked them whether it was acceptable to act. In all these cases, the wording indicated that people should give their own personal views about the acceptability of acting. In contrast, in our experiments, we asked participants to assess how many people out of 100 would consider acting acceptable. This was a question that was explicitly concerned with the social acceptability rather the personal acceptability of acting.

We had assumed that people’s personal views on the ethical acceptability of an action generally conform with their views of the social acceptability of carrying out that action. As a result, our experiment measuring the social acceptability of an ethical decision would broadly replicate the findings that have previously been reported in studies measuring the personal acceptability of an ethical decision. In other words, judgments of the social acceptability of ethical choices would be less utilitarian than the choices themselves (H_3_). Instead, we found that previous findings were reversed in our study: people’s ethical choices were less utilitarian than their judgments of the ethical acceptability of those choices.

What could explain this reversed pattern of results? Deontologists are considered to be nicer, more trustworthy, and more cooperative people than those holding to utilitarianism (Everett et al., 2016; Kahane et al., 2015). Thus, for egotistical reasons, people may consider themselves to be more deontological than other people. As a result, what they consider to be personally acceptable is likely to be influenced more by deontological considerations (previous studies) than what they consider to be socially acceptable (this study). Consequently, the judgments of the social acceptability of acting that we found here were more utilitarian than judgments of the personal acceptability of acting previously found by others (Gold et al., 2015; Kurzban et al., 2012; Tassy et al., 2013). This provides a possible explanation of the reversal of the effect of measurement type that we observed.

There is evidence supporting this argument. In the ‘actor’ version of Gold et al.’s (2015) paradigm that we have discussed up to this point, participants were asked to assess (a) whether they would act and (b) how right any such action of theirs would be. We have seen that, in this version of the paradigm, they found that the percentage of people in favor of acting was greater than the percentage of them judging the action to be permissible (i.e., not wrong). However, they also studied an ‘observer’ version of their paradigm in which participants were asked to assess (a) whether another person should act and (b) how right any such action by that other person would be. In this ‘observer’ version of the paradigm, the percentage of people in favor of acting was either *less* than or much the same as the percentage of them judging the action to be permissible. This difference between the two versions of their paradigm arose because people judged the rightness of an action performed by someone else to be greater than the rightness of the same action performed by themselves. In other words, their assessments of the acceptability of other people’s actions were more utilitarian than their assessments of the acceptability of the same actions performed by themselves. They were more deontological in the way that they viewed their own actions than in the way they viewed the same actions carried out by other people. Similar findings have been reported by Nadelhoffer and Feltz (2008).

We have suggested that such results occur because deontological approaches to ethical dilemmas are associated with desirable qualities (trustworthiness, cooperation) and people see themselves as being more likely than other people to exhibit those desirable qualities. However, there are other ways of explaining this pattern of results. Gold et al. (2015) propose that, because of the Fundamental Attribution Error (Ross and Nisbett, 1991), people consider other people to have more control over situations than they have themselves and, because of this, it is more acceptable for those other people to act than for themselves to act.

#### Cultural differences

Our sample of Chinese participants in Experiment 2 produced exactly the same pattern of results as the sample of English participants that we examined in Experiment 1. This is reassuring: our findings, even where unexpected, are robust. However, visual comparison of Figures 1, 2 suggests that at least some of the ratings were lower for the Chinese sample (range of means: 20–55) than for the English sample (range of means: 25–62). Previous findings have shown that East Asian people tend to adopt a more deontological approach to resolving ethical dilemmas than Western people (e.g., Ahlenius and Tännsjö, 2012; Awad et al., 2018; Costa et al., 2014; Gold et al., 2014).

A four-way mixed model ANOVA using the same three within-participant factors as before but adding culture (Experiment 1 versus Experiment 2) as a between-participant factor revealed significant main effects of action type (*F*(1, 186) = 99.24, *p* < 0.001, *η^2^* = 0.069) and measurement type (*F*(1, 186) = 137.11, *p* < 0.001, *η^2^* = 0.081). These findings were to be expected as both effects were present in the separate analyses of each experiment. The main effect of culture did not reach significance (*F*(1, 186) = 3.2, *p* = 0.075, *η^2^* = 0.010) but two interactions with culture were significant.

The first significant interaction was between culture and measurement type (*F*(1, 186) = 4.18, *p* = 0.042, *η^2^* = 0.003). Analysis of simple effects showed this arose because English participants made significantly more utilitarian decisions about whether to act (*t*(743.85) = 3.81, *p* < 0.001, *d* = 0.278) but not about the social acceptability of acting (*t*(743.35) = 1.34, *p* = 0.182, *d* = 0.097). As a result, the differences between ratings for acting and the social acceptability of acting were greater for Chinese than for English participants. (Compare Figures 1, 2.)

Despite the fact that there was no significant difference in judgments of the social acceptability of sacrificial action across cultures, Chinese people were significantly less likely to select a sacrificial action themselves than English people were. What can explain this? Whereas people from both cultures judged that about half of their compatriots would carry out a sacrificial action, Chinese people may have *cared* more about the people who disagreed with such action. Previous researchers have made this suggestion. For example, Gold et al. (2014, p. 74) argued that Chinese people’s “reluctance to act may be exacerbated by the fact that Chinese have more inter-dependent self-construals, one consequence of which is that they care more about the opinions of others (Markus and Kitayama, 1991). The Chinese may have been more worried about being negatively perceived by others if they caused harm to someone when taking a decision that they felt they had no right to make.” In other words, Chinese people are more sensitive than English people to the social acceptability of making a sacrificial action.

This suggests that we should distinguish people’s perceptions of the social acceptability of a sacrificial action (assessed by their judgments of the proportion of their compatriots who would carry it out) and how sensitive those people are to their perceptions of social acceptability. In other words, when making an ethical decision, people are influenced by (a) their assessments of the inherent rightness of different courses of action, and (b) their assessments of the proportions of people who would select each of those courses of action. In some (e.g., Chinese) cultures, more weight is given to this second (social acceptability) factor. Clearly, these ideas need to be elaborated and tested; they are currently speculative. A measure of the influence of people’s assessment of the social acceptability of a sacrificial action on their judgments of whether to take such an action needs to be developed. Regression models could then be formulated and compared to cast more light on how ethical judgments are made.

The second significant interaction was between culture and action type (*F* (1, 186) = 5.71, *p* = 0.018, *η^2^* = 0.004). Analysis of simple effects showed that Chinese participants made significantly more utilitarian judgments for side-track dilemmas (*t* (749.87) = 4.31, *p* < 0.001, *d* = 0.314) but not for footbridge dilemmas (*t* (748.23) = 0.912, *p* = 0.362, *d* = 0.066). Others have found similar results: Ahlenius and Tännsjö (2012) found a large cultural difference in utilitarian responding in the side-track dilemma with 81% of Americans choosing to act but only 52% of Chinese people doing so. In contrast, while they found a cultural difference in footbridge dilemma, it was much smaller with 39% of Americans choosing to act but 22% of Chinese people doing so.

Findings showing that experimental manipulations affect responses to personal but not impersonal dilemmas (or vice versa) have been typically explained in terms of Greene et al.’s (2001) dual system model (e.g., Costa et al., 2014). Our findings can be explained in the same way. We assume that both Chinese and English participants respond to footbridge dilemmas using the intuitive/emotional processing provided by System 1, just as Greene et al. (2001) propose. However, whereas English participants typically use the deliberative processing provided by System 2 to respond to side-track dilemmas, many Chinese participants engage in System 1 emotional processing even when addressing these dilemmas. Gold et al. (2014) found that the modal rationale given by Chinese participants for not acting in the side-track scenario was one of ‘not having the right to intervene’. This implies that they may have been emotionally torn between a personal desire to act and an awareness of social constraints forbidding them to do so.

## General discussion

The pattern of results that we obtained can be interpreted within a coherent framework. The key to achieving this is in recognizing a distinction that has not been fully appreciated in the past. In previous studies, people have been asked to use a Likert scale to judge how right a sacrificial action would be (Gold et al., 2014), to make a binary decision on the acceptability of a sacrificial action (Tassy et al., 2013), or to make a binary decision about whether a sacrificial action is morally wrong (Kurzban et al., 2012). In all these cases, the wording implied that people were to judge whether a sacrificial action would be *personally* acceptable to them. In contrast, in our experiments, participants were required to assess how many people out of 100 would judge the sacrificial action to be ethically acceptable. In other words, they were to judge the degree to which the action would be *socially* acceptable. This difference in task requirements can explain why the direction of the effect of measurement type that we obtained was different from that reported in previous studies.

Previous work has shown that, on average, people are *more* willing to act in a utilitarian manner than they are to judge that acting in such a manner is ethically acceptable to them personally. Such behavior implies that, when deciding to act, people take into account not just how ethically acceptable they judge acting to be but also additional factors (e.g., how much not acting would prompt feelings of regret).

People’s judgments were more deontological than they would be expected to be if they were making them purely on the basis of their perceived social acceptability of a sacrificial action. We suggested above that this is because people prefer to act in a more deontological way than other people because acting in a deontological (principled) manner is seen as a desirable trait. But what could explain the fact that this tendency toward deontology is even greater in Chinese participants? It could be that acting in a deontological manner is seen as even more desirable in Chinese people. Alternatively, there may be other factors that drive people toward a deontological approach and these factors were more influential, stronger, or more numerous in Chinese people. What could such factors be? One possibility is that Chinese people are more concerned about the possibility of being blamed for acting against social norms than for not acting when social norms favor acting.

Potential for blame is likely to be an especially important factor to take into consideration in societies where it is associated with negative consequences (e.g., ostracism). Gold et al.’s (2014) finding that Chinese participants were most likely to explain their failure to act in the side-track dilemma as not having the right to intervene can be seen as expressing a concern that they will be blamed if they do intervene. As Gold et al. (2014, p. 74) go on to point out, ‘Chinese have more inter-dependent self-construals, one consequence of which is that they care more about the opinion of others (Markus and Kitayama, 1991)’. This can explain why we found that Chinese participants showed more difference than English participants between how utilitarian they were when judging whether to act and how utilitarian they were when assessing the social acceptability of acting. Both samples showed a similar degree of social acceptability of acting but, in choosing whether to act themselves, Chinese people were more sensitive to the fact that many of their compatriots would choose not to act.

Why are East Asian people less likely than Western people to act in a utilitarian manner in the side-track dilemma but not in the footbridge dilemma? Greene et al. (2001) argue that lower utilitarian responding in the footbridge dilemma than in the side-track dilemma occurs because its personal nature engages emotions that are processed by System 1 and, hence, produces a deontological resolution to the dilemma. We assume that this occurs across all cultures. Greene et al. (2001) also argue that responding to the impersonal side-track dilemma does not engage emotions and relies on the deliberative processing provided by System 2. However, this may be true only of Western participants. Greater concern about the opinion of others, particularly the potential for being blamed for acting in sacrificial dilemmas, implies that East Asians are more likely to anticipate feelings of regret when considering such action. Processing such feelings will require engagement of System 1 even when addressing the side-track dilemma. As a result, East Asians are less likely than Western people to respond in a utilitarian manner in the side-track scenario.

Thus, the fact that we specifically required participants to assess the social rather than the personal acceptability of acting in a utilitarian manner can explain (a) why the effect of measurement type was the reverse of what has been previously found, (b) why our Chinese participants showed more difference than English participants between how utilitarian they were when judging whether to act and how utilitarian they were when assessing the social acceptability of acting, and (c) why Chinese participants were less likely than English participants to respond in a utilitarian manner in the side-track dilemma but equally likely to do so in the footbridge dilemma.

### Limitations and future work

Use of trolley dilemmas has been criticized in the past for being a contrived and unrealistic way of studying how people make ethical judgments (Bauman et al., 2014). However, since that criticism was aired, more realistic versions of trolley dilemmas have replicated earlier findings (e.g., Awad et al., 2018; Bonnefon et al., 2016; Christen et al., 2021; Meder et al., 2019). For recent reviews on advantages and disadvantages of using trolley dilemmas in research into ethical judgment, see Lillehammer (2023).

Our participants were culturally English (Experiment 1) or culturally Chinese (Experiment 2). We would expect that members of these two groups are broadly representative of Western and East Asian people, respectively (c.f., Nisbett, 2003). However, it is important to bear in mind that there are some reports of differences in the way that individuals within each of these two broad groupings make judgments. For example, Yates et al. (1989) showed that Japanese people made probability judgments in the same way as American people but that people in both of these groups made these judgments in a different way from Chinese people.

Demographic characteristics of participants in the two samples varied somewhat: people within the Chinese sample were younger, more educated and a greater proportion of them were women. However, the main effects of measurement type were obtained in both samples and it is unclear how demographic differences could account for the cultural differences that we report. Both samples were solicited via Prolific or via snowball sampling (i.e., those who had already taken part in the experiment via one of these channels encouraged their acquaintances to take part). While no method for soliciting experimental participants is perfect, empirical comparison has shown that, relative to other sites, Prolific provides participants that are ‘more likely to pass various attention checks, provide meaningful answers, follow instructions, remember previously presented information, have a unique IP address and geolocation, and to work slowly enough to be able to read all the items’ (Douglas et al., 2023, p1).

Central to interpretation of our findings is the suggestion that the effects of personal acceptability of ethical choices and the effects of people’s judgments about the social acceptability of those choices are separate and distinct. They are affected by different factors and they affect ethical choices in different ways. In future work, it will be important to examine the effects of both of them in the same study.

In future, it would also be sensible to study just how people take into account their assessments of the social acceptability of different courses of action when they make ethical choices. People in some cultures may be more sensitive to their estimates of the social acceptability of sacrificial action than those in others. In our view, people have an idea of what the ‘best’ or the ‘right’ thing to do is. However, they have to reconcile that with their judgment of what is likely to be acceptable within their society. What they judge to be right and what they judge to be acceptable may not differ. But, if they do, they must either select between them or weight them in some way to produce a compromise. Our results suggest that the way that they weight them may be culture-dependent.

### Implications

It is now recognized that the ethical dilemmas investigated via trolley scenarios are present in many real situations, such as in programming of autonomous vehicles (Awad et al., 2018; Bonnefon et al., 2016; Meder et al., 2019) and in drone warfare (Christen et al., 2021). Cultural differences, such as those uncovered here, imply that the way that these real dilemmas are resolved is likely to depend on the cultural context in which they appear.

## Data Availability

The datasets presented in this study can be found in online repositories. The names of the repository/repositories and accession number(s) can be found at: Open Science Framework: https://osf.io/9qcus/?view_only=089b0aeb32bb4e44bc913a61d6cdbe80.

## Footnotes

^1^In Foot’s original version, the driver of a runaway tram makes the decision rather than an agent outside the tram.

^2^For a discussion of how psychologists came to use trolley dilemmas as a means for determining whether people act in a deontological or utilitarian manner, see Kahane and Everett (2023).

^3^There are many other versions of the trolley dilemma besides the switch and footbridge scenarios, including the loop and bridge collapse scenarios (Mikhail, 2009).

^4^ www.prolific.co

^5^A Shapiro–Wilk test showed the non-normal distribution of the responses (*W* = 0.918, *p* < 0.001) but, with a sample size as large as ours, this is unlikely to limit the robustness of the ANOVA (Field et al., 2012).

^6^A Shapiro–Wilk test showed the non-normal distribution of the responses (*W* = 0.918, *p* < 0.001) but, with a sample size as large as ours, this is unlikely to limit the robustness of the ANOVA (Field et al., 2012).

^7^To exclude any influence of answering the question about social acceptability of sacrificial action before the question about the likelihood of performing that action, our experiments could be repeated with the questions presented in the reverse order or with separate groups of people answering each question.
